# Ethyl 4-(1,3-benzodioxol-5-yl)-6-methyl-2-sulfanylidene-1,2,3,4-tetra­hydro­pyrimidine-5-carboxyl­ate

**DOI:** 10.1107/S1600536811043649

**Published:** 2011-10-29

**Authors:** Susanta K. Nayak, K. N. Venugopala, Thavendran Govender, Hendrik G. Kruger, Glenn E. M. Maguire, Tayur N. Guru Row

**Affiliations:** a Solid State and Structural Chemistry Unit, Indian Institute of Science, Bangalore 560 012, India; b School of Chemistry, University of KwaZulu-Natal, Durban 4000, South Africa; c School of Pharmacy and Pharmacology, University of KwaZulu-Natal, Durban 4000, South Africa

## Abstract

In the title compound, C_15_H_16_N_2_O_4_S, the dihedral angles between the planes of the benzodioxole and ester groups and the plane of the six-membered tetra­hydro­pyrimidine ring are 89.5 (1) and 20.2 (1)°, respectively. Inter­molecular N—H⋯S hydrogen bonds assemble the mol­ecules into dimers, which are further connected *via* N—H⋯O inter­actions into chains parallel to [010]. Weak C—H⋯S and C—H⋯π inter­actions enhance the stability of the crystal structure.

## Related literature

For background to the applications of multi-functionalized dihydro­pyrimidines, see: Jauk *et al.* (2000 ▶); Kappe (2000 ▶); Mayer *et al.* (1999 ▶). For similar structures, see: Nayak *et al.* (2009 ▶, 2010 ▶, 2011 ▶).
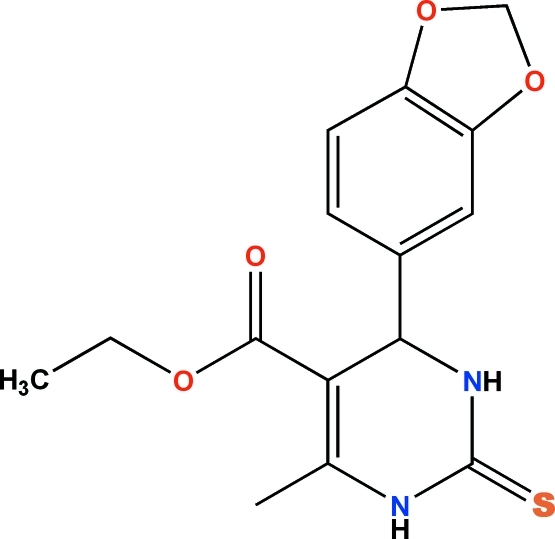


## Experimental

### 

#### Crystal data

C_15_H_16_N_2_O_4_S
*M* *_r_* = 320.37
Monoclinic, 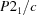

*a* = 12.5102 (9) Å
*b* = 7.2054 (4) Å
*c* = 17.0881 (12) Å
β = 107.178 (8)°
*V* = 1471.62 (17) Å^3^
*Z* = 4
Mo *K*α radiation
μ = 0.24 mm^−1^
*T* = 120 K
0.28 × 0.22 × 0.19 mm

#### Data collection

Oxford Diffraction Xcalibur E diffractometer
Absorption correction: multi-scan (*CrysAlis PRO*; Oxford Diffraction, 2009 ▶) *T*_min_ = 0.936, *T*_max_ = 0.956
18163 measured reflections
2883 independent reflections
2349 reflections with *I* > 2σ(*I*)
*R*_int_ = 0.067

#### Refinement

*R*[*F* ^2^ > 2σ(*F* ^2^)] = 0.038
*wR*(*F* ^2^) = 0.103
*S* = 1.09
2883 reflections
263 parameters
All H-atom parameters refined
Δρ_max_ = 0.33 e Å^−3^
Δρ_min_ = −0.30 e Å^−3^

### 

Data collection: *CrysAlis PRO* (Oxford Diffraction, 2009 ▶); cell refinement: *CrysAlis PRO*; data reduction: *CrysAlis PRO*; program(s) used to solve structure: *SHELXS97* (Sheldrick, 2008 ▶); program(s) used to refine structure: *SHELXL97* (Sheldrick, 2008 ▶); molecular graphics: *ORTEP-3 for Windows* (Farrugia, 1997 ▶) and *CAMERON* (Watkin *et al.*, 1993 ▶); software used to prepare material for publication: *PLATON* (Spek, 2009 ▶) and *PARST* (Nardelli, 1995 ▶).

## Supplementary Material

Crystal structure: contains datablock(s) global, I. DOI: 10.1107/S1600536811043649/gk2420sup1.cif

Structure factors: contains datablock(s) I. DOI: 10.1107/S1600536811043649/gk2420Isup2.hkl

Supplementary material file. DOI: 10.1107/S1600536811043649/gk2420Isup3.cml

Additional supplementary materials:  crystallographic information; 3D view; checkCIF report

## Figures and Tables

**Table 1:** Hydrogen-bond geometry (Å, °) *Cg*1 and *Cg*2 are the centroids of the O3/C11/C12/O5/C15 and  C9–C14 rings respectively.

*D*—H⋯*A*	*D*—H	H⋯*A*	*D*⋯*A*	*D*—H⋯*A*
N1—H1N⋯O1^i^	0.84 (2)	2.14 (2)	2.9578 (19)	167 (2)
N2—H2N⋯S1^ii^	0.76 (2)	2.57 (2)	3.3069 (17)	164 (2)
C10—H10⋯S1^iii^	0.95 (2)	2.75 (2)	3.678 (2)	166.9 (18)
C15—H15*B*⋯*Cg*1^iv^	0.98 (3)	2.95 (3)	3.890 (2)	160 (2)
C6—H6*A*⋯*Cg*2^v^	0.98 (2)	2.87 (2)	3.691 (2)	142 (2)

Symmetry codes: (i) 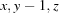; (ii) 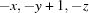; (iii) 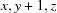; (iv) 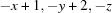; (v) 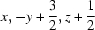.

**Fig. 1.**
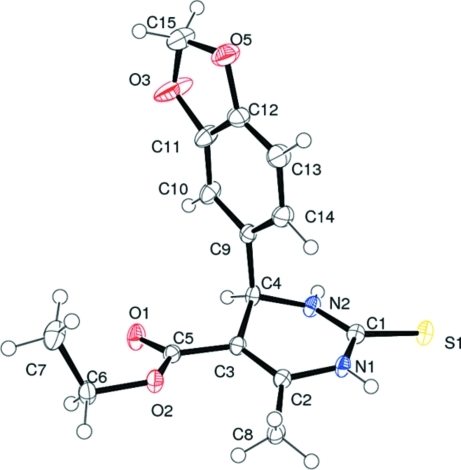
Molecular structure of the title compound with displacement ellipsoids for non-H atoms at the 50% probability level.

**Fig. 2.**
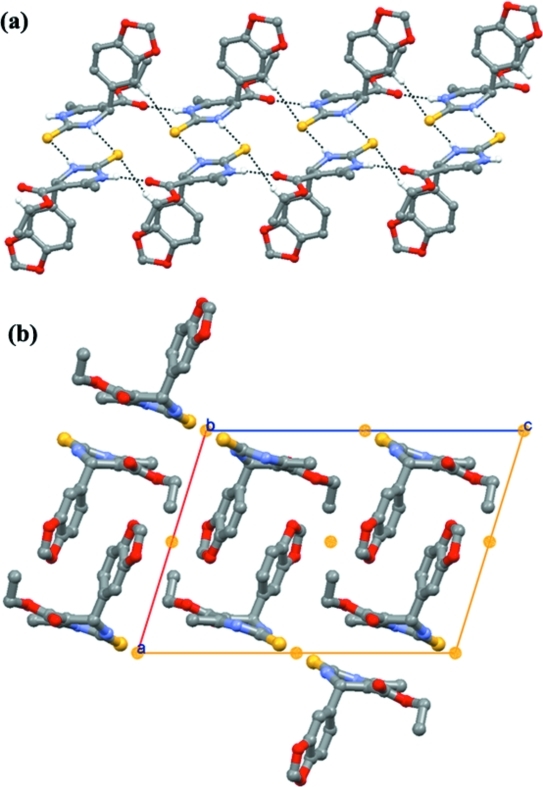
(a) The crystal packing diagram showing hydrogen-bonded chains formed by hydrogen-bonded dimers. (b) View of the crystal packing along the b axis (yellow circle represents centre of inversion).

## supplementary crystallographic information

### Comment

Biginelli compounds are multifunctionalized dihydropyrimidones (DHPM) (Kappe, 2000 and references therein), which have emerged as important target molecule for therapeutic and pharmacological properties such as anticarcinogenic (Kappe, 2000; Mayer *et al.*, 1999) and calcium channel modulators (Jauk *et al.*, 2000 and references therein). In view of the immense range of applications, we became interested in the DHPMS system (Nayak *et al.*,2009; 2010; 2011). Here we report a single-crystal structure of the title compound.

The preferred conformation of the molecule is that with the dihedral angles between the planes of benzodioxolyl and ester groups (O1/C5/O2/C6/C7) with the plane of the six-membered tetrahydropyrimidine ring of 89.5 (1) ° and 20.2 (1) °, respectively. The centrosymmetric N—H···S dimers of the title molecules are additionally organized into chains along the *b* axis via N—H···O hydrogen bonds. These chains are also stabilized by weak C—H···S interactions (Fig. 2). Additonal weak C—H···π interactions enhance the crystal stability.

### Experimental

A mixture of ethyl acetoacetate (0.1 mol), 3,4-(methylenedioxy)benzaldehyde (0.1 mol) and thiourea (0.1 mol) was refluxed in 50 ml of ethanol for 2 h in the presence of concentrated hydrochloric acid as a catalyst. The reaction was monitored with thin layer chromatography and the reaction medium was quenched in ice cold water. The precipitate obtained was filtered, dried and crystallized from methanol at room temperature to obtain the title compound.

### Refinement

All H atoms were located from difference Fourier map and refined freely.

### Crystal data

**Table:**

C_15_H_16_N_2_O_4_S	*F*(000) = 672
*M**_r_* = 320.37	*D*_x_ = 1.446 Mg m^−^^3^
Monoclinic, *P*2_1_/*c*	Melting point: 447(2) K
Hall symbol: -P 2ybc	Mo *K*α radiation, λ = 0.71073 Å
*a* = 12.5102 (9) Å	Cell parameters from 430 reflections
*b* = 7.2054 (4) Å	θ = 1.0–27.9°
*c* = 17.0881 (12) Å	µ = 0.24 mm^−^^1^
β = 107.178 (8)°	*T* = 120 K
*V* = 1471.62 (17) Å^3^	Block, colorless
*Z* = 4	0.28 × 0.22 × 0.19 mm

### Data collection

**Table:**

Oxford Diffraction Xcalibur E diffractometer	2883 independent reflections
Radiation source: Enhance (Mo) X-ray Source	2349 reflections with *I* > 2σ(*I*)
graphite	*R*_int_ = 0.067
Detector resolution: 16.0839 pixels mm^-1^	θ_max_ = 26.0°, θ_min_ = 2.5°
ω scans	*h* = −15→15
Absorption correction: multi-scan (*CrysAlis PRO*; Oxford Diffraction, 2009)	*k* = −8→8
*T*_min_ = 0.936, *T*_max_ = 0.956	*l* = −21→21
18163 measured reflections	

### Refinement

**Table:**

Refinement on *F*^2^	Primary atom site location: structure-invariant direct methods
Least-squares matrix: full	Secondary atom site location: difference Fourier map
*R*[*F*^2^ > 2σ(*F*^2^)] = 0.038	Hydrogen site location: inferred from neighbouring sites
*wR*(*F*^2^) = 0.103	All H-atom parameters refined
*S* = 1.09	*w* = 1/[σ^2^(*F*_o_^2^) + (0.044*P*)^2^ + 0.4413*P*] where *P* = (*F*_o_^2^ + 2*F*_c_^2^)/3
2883 reflections	(Δ/σ)_max_ < 0.001
263 parameters	Δρ_max_ = 0.33 e Å^−^^3^
0 restraints	Δρ_min_ = −0.30 e Å^−^^3^

### Special details

**Table:**

Geometry. All s.u.'s (except the s.u. in the dihedral angle between two l.s. planes) are estimated using the full covariance matrix. The cell s.u.'s are taken into account individually in the estimation of s.u.'s in distances, angles and torsion angles; correlations between s.u.'s in cell parameters are only used when they are defined by crystal symmetry. An approximate (isotropic) treatment of cell s.u.'s is used for estimating s.u.'s involving l.s. planes.
Refinement. Refinement of *F*^2^ against ALL reflections. The weighted *R*-factor *wR* and goodness of fit *S* are based on *F*^2^, conventional *R*-factors *R* are based on *F*, with *F* set to zero for negative *F*^2^. The threshold expression of *F*^2^ > 2σ(*F*^2^) is used only for calculating *R*-factors(gt) *etc*. and is not relevant to the choice of reflections for refinement. *R*-factors based on *F*^2^ are statistically about twice as large as those based on *F*, and *R*- factors based on ALL data will be even larger.

### Fractional atomic coordinates and isotropic or equivalent isotropic displacement parameters (Å^2^)

**Table:**

	*x*	*y*	*z*	*U*_iso_*/*U*_eq_	
S1	0.03990 (4)	0.22578 (6)	0.06240 (3)	0.02136 (16)	
O2	0.21637 (11)	0.77438 (16)	0.39149 (7)	0.0190 (3)	
C4	0.14810 (16)	0.7167 (2)	0.17046 (11)	0.0167 (4)	
O1	0.14472 (12)	0.97853 (17)	0.29048 (8)	0.0246 (3)	
C9	0.26335 (16)	0.7523 (2)	0.16168 (10)	0.0164 (4)	
N1	0.12326 (13)	0.3507 (2)	0.21437 (9)	0.0178 (4)	
N2	0.08802 (14)	0.5707 (2)	0.11385 (10)	0.0179 (4)	
C2	0.14299 (15)	0.4837 (2)	0.27642 (11)	0.0161 (4)	
C5	0.16897 (15)	0.8200 (2)	0.31311 (11)	0.0164 (4)	
C3	0.15224 (15)	0.6627 (2)	0.25676 (10)	0.0157 (4)	
C8	0.14782 (18)	0.4050 (3)	0.35838 (12)	0.0194 (4)	
C1	0.08656 (15)	0.3933 (2)	0.13312 (11)	0.0173 (4)	
C6	0.23338 (18)	0.9229 (3)	0.45154 (12)	0.0214 (4)	
C12	0.47322 (16)	0.8166 (3)	0.14736 (12)	0.0242 (4)	
C10	0.28717 (17)	0.9248 (3)	0.13323 (12)	0.0240 (4)	
O5	0.57092 (12)	0.8812 (2)	0.13570 (10)	0.0348 (4)	
C14	0.34567 (17)	0.6153 (3)	0.18099 (12)	0.0236 (4)	
C13	0.45264 (18)	0.6459 (3)	0.17449 (13)	0.0268 (5)	
O3	0.43410 (14)	1.1070 (2)	0.09967 (13)	0.0551 (6)	
C11	0.39210 (18)	0.9510 (3)	0.12682 (13)	0.0267 (5)	
C7	0.3423 (2)	1.0191 (3)	0.46053 (15)	0.0316 (5)	
C15	0.54868 (19)	1.0701 (3)	0.10926 (16)	0.0341 (5)	
H8A	0.1201 (17)	0.491 (3)	0.3892 (13)	0.022 (5)*	
H6B	0.1710 (17)	1.006 (3)	0.4352 (12)	0.017 (5)*	
H10	0.2310 (19)	1.017 (3)	0.1214 (13)	0.030 (6)*	
H8B	0.1075 (18)	0.292 (3)	0.3532 (12)	0.022 (5)*	
H13	0.5136 (19)	0.551 (3)	0.1892 (13)	0.030 (6)*	
H2N	0.0633 (19)	0.599 (3)	0.0693 (14)	0.023 (6)*	
H1N	0.122 (2)	0.240 (3)	0.2286 (14)	0.031 (7)*	
H4	0.1015 (16)	0.829 (3)	0.1527 (11)	0.015 (5)*	
H6A	0.2297 (16)	0.861 (3)	0.5017 (12)	0.016 (5)*	
H14	0.326 (2)	0.496 (3)	0.2002 (14)	0.042 (7)*	
H7A	0.408 (2)	0.932 (4)	0.4772 (16)	0.050 (7)*	
H7B	0.3470 (19)	1.077 (3)	0.4079 (14)	0.033 (6)*	
H8C	0.222 (2)	0.379 (3)	0.3889 (15)	0.038 (7)*	
H15A	0.593 (2)	1.155 (4)	0.1543 (16)	0.048 (7)*	
H7C	0.349 (2)	1.111 (4)	0.5042 (16)	0.051 (8)*	
H15B	0.556 (2)	1.086 (4)	0.0541 (17)	0.053 (8)*	

### Atomic displacement parameters (Å^2^)

**Table:**

	*U*^11^	*U*^22^	*U*^33^	*U*^12^	*U*^13^	*U*^23^
S1	0.0238 (3)	0.0142 (3)	0.0213 (3)	0.00006 (18)	−0.0006 (2)	−0.00166 (17)
O2	0.0258 (8)	0.0137 (7)	0.0154 (6)	−0.0003 (5)	0.0032 (6)	−0.0012 (5)
C4	0.0206 (10)	0.0107 (9)	0.0162 (9)	0.0004 (7)	0.0014 (7)	−0.0004 (7)
O1	0.0350 (8)	0.0129 (7)	0.0225 (7)	0.0038 (6)	0.0033 (6)	0.0012 (5)
C9	0.0188 (10)	0.0162 (9)	0.0129 (8)	−0.0021 (7)	0.0023 (7)	−0.0014 (7)
N1	0.0230 (9)	0.0095 (8)	0.0194 (8)	0.0010 (6)	0.0040 (7)	0.0005 (6)
N2	0.0211 (9)	0.0136 (8)	0.0157 (9)	−0.0001 (6)	0.0004 (7)	0.0027 (6)
C2	0.0143 (9)	0.0145 (9)	0.0188 (9)	0.0018 (7)	0.0037 (7)	0.0009 (7)
C5	0.0159 (9)	0.0144 (9)	0.0194 (9)	0.0008 (7)	0.0060 (7)	−0.0001 (7)
C3	0.0159 (9)	0.0139 (9)	0.0162 (9)	0.0008 (7)	0.0033 (7)	0.0003 (7)
C8	0.0258 (12)	0.0126 (10)	0.0199 (10)	0.0005 (8)	0.0067 (9)	0.0011 (7)
C1	0.0127 (9)	0.0169 (10)	0.0205 (9)	0.0017 (7)	0.0020 (7)	−0.0002 (7)
C6	0.0301 (12)	0.0165 (10)	0.0164 (9)	−0.0008 (8)	0.0050 (8)	−0.0028 (7)
C12	0.0177 (10)	0.0290 (11)	0.0262 (10)	−0.0032 (8)	0.0070 (8)	−0.0042 (8)
C10	0.0209 (11)	0.0165 (10)	0.0321 (11)	0.0008 (8)	0.0040 (9)	0.0037 (8)
O5	0.0236 (8)	0.0302 (9)	0.0538 (10)	−0.0040 (6)	0.0162 (7)	0.0021 (7)
C14	0.0266 (11)	0.0175 (10)	0.0283 (10)	0.0020 (8)	0.0102 (9)	0.0049 (8)
C13	0.0232 (11)	0.0219 (11)	0.0360 (11)	0.0047 (8)	0.0099 (9)	0.0034 (9)
O3	0.0303 (10)	0.0329 (10)	0.1070 (16)	−0.0001 (7)	0.0281 (10)	0.0314 (10)
C11	0.0264 (11)	0.0187 (10)	0.0345 (11)	−0.0037 (8)	0.0083 (9)	0.0061 (8)
C7	0.0301 (13)	0.0261 (12)	0.0348 (13)	−0.0056 (10)	0.0036 (10)	−0.0075 (10)
C15	0.0274 (13)	0.0331 (13)	0.0427 (14)	−0.0071 (9)	0.0117 (11)	0.0052 (10)

### Geometric parameters (Å, °)

**Table:**

S1—C1	1.6854 (18)	C8—H8C	0.94 (3)
O2—C5	1.336 (2)	C6—C7	1.496 (3)
O2—C6	1.454 (2)	C6—H6B	0.96 (2)
C4—N2	1.476 (2)	C6—H6A	0.98 (2)
C4—C3	1.511 (2)	C12—C13	1.365 (3)
C4—C9	1.515 (3)	C12—C11	1.372 (3)
C4—H4	0.99 (2)	C12—O5	1.376 (2)
O1—C5	1.215 (2)	C10—C11	1.362 (3)
C9—C14	1.394 (3)	C10—H10	0.94 (2)
C9—C10	1.398 (3)	O5—C15	1.435 (3)
N1—C1	1.362 (2)	C14—C13	1.393 (3)
N1—C2	1.396 (2)	C14—H14	0.97 (2)
N1—H1N	0.84 (2)	C13—H13	1.00 (2)
N2—C1	1.322 (2)	O3—C11	1.378 (2)
N2—H2N	0.76 (2)	O3—C15	1.419 (3)
C2—C3	1.346 (2)	C7—H7A	1.01 (3)
C2—C8	1.495 (3)	C7—H7B	1.01 (2)
C5—C3	1.462 (2)	C7—H7C	0.98 (3)
C8—H8A	0.94 (2)	C15—H15A	1.01 (3)
C8—H8B	0.95 (2)	C15—H15B	0.98 (3)
			
C5—O2—C6	117.10 (14)	O2—C6—H6B	108.5 (12)
N2—C4—C3	108.68 (15)	C7—C6—H6B	112.3 (12)
N2—C4—C9	111.78 (15)	O2—C6—H6A	104.3 (11)
C3—C4—C9	112.39 (15)	C7—C6—H6A	113.5 (11)
N2—C4—H4	104.0 (11)	H6B—C6—H6A	107.2 (17)
C3—C4—H4	110.9 (11)	C13—C12—C11	121.57 (19)
C9—C4—H4	108.7 (11)	C13—C12—O5	128.22 (19)
C14—C9—C10	119.55 (18)	C11—C12—O5	110.21 (18)
C14—C9—C4	120.99 (17)	C11—C10—C9	117.43 (18)
C10—C9—C4	119.46 (16)	C11—C10—H10	124.0 (14)
C1—N1—C2	123.39 (16)	C9—C10—H10	118.6 (14)
C1—N1—H1N	118.8 (16)	C12—O5—C15	105.58 (16)
C2—N1—H1N	116.7 (16)	C13—C14—C9	121.93 (19)
C1—N2—C4	124.61 (16)	C13—C14—H14	120.6 (15)
C1—N2—H2N	118.7 (17)	C9—C14—H14	117.4 (15)
C4—N2—H2N	116.1 (17)	C12—C13—C14	116.87 (18)
C3—C2—N1	118.48 (16)	C12—C13—H13	119.6 (13)
C3—C2—C8	127.90 (17)	C14—C13—H13	123.6 (13)
N1—C2—C8	113.59 (15)	C11—O3—C15	106.32 (17)
O1—C5—O2	123.07 (16)	C10—C11—C12	122.62 (19)
O1—C5—C3	123.06 (16)	C10—C11—O3	127.79 (19)
O2—C5—C3	113.85 (15)	C12—C11—O3	109.59 (18)
C2—C3—C5	125.78 (16)	C6—C7—H7A	112.5 (15)
C2—C3—C4	120.63 (16)	C6—C7—H7B	113.0 (13)
C5—C3—C4	113.58 (15)	H7A—C7—H7B	105 (2)
C2—C8—H8A	110.8 (12)	C6—C7—H7C	104.9 (15)
C2—C8—H8B	111.5 (12)	H7A—C7—H7C	109 (2)
H8A—C8—H8B	110.1 (18)	H7B—C7—H7C	112.7 (19)
C2—C8—H8C	110.8 (15)	O3—C15—O5	108.02 (17)
H8A—C8—H8C	106.6 (19)	O3—C15—H15A	106.3 (15)
H8B—C8—H8C	106.9 (19)	O5—C15—H15A	108.9 (15)
N2—C1—N1	116.51 (16)	O3—C15—H15B	104.3 (16)
N2—C1—S1	122.78 (14)	O5—C15—H15B	110.3 (16)
N1—C1—S1	120.71 (14)	H15A—C15—H15B	118 (2)
O2—C6—C7	110.65 (17)		
			
N2—C4—C9—C14	−64.5 (2)	C4—N2—C1—S1	−167.04 (14)
C3—C4—C9—C14	58.0 (2)	C2—N1—C1—N2	11.3 (3)
N2—C4—C9—C10	115.08 (18)	C2—N1—C1—S1	−167.60 (15)
C3—C4—C9—C10	−122.40 (18)	C5—O2—C6—C7	−86.5 (2)
C3—C4—N2—C1	−30.0 (2)	C14—C9—C10—C11	−0.9 (3)
C9—C4—N2—C1	94.6 (2)	C4—C9—C10—C11	179.58 (18)
C1—N1—C2—C3	−16.3 (3)	C13—C12—O5—C15	−178.2 (2)
C1—N1—C2—C8	162.00 (17)	C11—C12—O5—C15	2.3 (2)
C6—O2—C5—O1	3.2 (3)	C10—C9—C14—C13	1.4 (3)
C6—O2—C5—C3	−178.61 (16)	C4—C9—C14—C13	−179.05 (18)
N1—C2—C3—C5	177.79 (17)	C11—C12—C13—C14	−0.5 (3)
C8—C2—C3—C5	−0.2 (3)	O5—C12—C13—C14	179.97 (19)
N1—C2—C3—C4	−3.5 (3)	C9—C14—C13—C12	−0.7 (3)
C8—C2—C3—C4	178.49 (18)	C9—C10—C11—C12	−0.3 (3)
O1—C5—C3—C2	−157.7 (2)	C9—C10—C11—O3	179.2 (2)
O2—C5—C3—C2	24.2 (3)	C13—C12—C11—C10	1.0 (3)
O1—C5—C3—C4	23.5 (3)	O5—C12—C11—C10	−179.37 (19)
O2—C5—C3—C4	−154.60 (16)	C13—C12—C11—O3	−178.55 (19)
N2—C4—C3—C2	23.6 (2)	O5—C12—C11—O3	1.0 (2)
C9—C4—C3—C2	−100.7 (2)	C15—O3—C11—C10	176.5 (2)
N2—C4—C3—C5	−157.57 (15)	C15—O3—C11—C12	−4.0 (3)
C9—C4—C3—C5	78.17 (19)	C11—O3—C15—O5	5.3 (3)
C4—N2—C1—N1	14.1 (3)	C12—O5—C15—O3	−4.7 (2)

### Hydrogen-bond geometry (Å, °)

**Table:**

*D*—H···*A*	*D*—H	H···*A*	*D*···*A*	*D*—H···*A*
N1—H1N···O1^i^	0.84 (2)	2.14 (2)	2.9578 (19)	167 (2)
N2—H2N···S1^ii^	0.76 (2)	2.57 (2)	3.3069 (17)	164 (2)
C10—H10···S1^iii^	0.95 (2)	2.75 (2)	3.678 (2)	166.9 (18)
C15—H15B···Cg1^iv^	0.98 (3)	2.95 (3)	3.890 (2)	160 (2)
C6—H6A···Cg2^v^	0.98 (2)	2.87 (2)	3.691 (2)	142 (2)

Symmetry codes: (i) *x*, *y*−1, *z*; (ii) −*x*, −*y*+1, −*z*; (iii) *x*, *y*+1, *z*; (iv) −*x*+1, −*y*+2, −*z*; (v) *x*, −*y*+3/2, *z*+1/2.
